# Relationships between childhood trauma and mental health during the COVID-19 pandemic: a network analysis

**DOI:** 10.3389/fpsyt.2023.1251473

**Published:** 2023-09-08

**Authors:** Jinjin Xia, Lin Zhu, Huayun Huang, Pengfei Fan, Meifeng Zhou, Xin-lu Cai, Hui He

**Affiliations:** ^1^Department of Neurology, Changxing People’s Hospital of Zhejiang Province, Huzhou, China; ^2^Department of Neurology, The Second Affiliated Hospital, Zhejiang University School of Medicine, Hangzhou, China; ^3^Institute of Brain Science and Department of Physiology, School of Basic Medical Sciences, Hangzhou Normal University, Hangzhou, China; ^4^Zhejiang Philosophy and Social Science Laboratory for Research in Early Development and Childcare, Hangzhou Normal University, Hangzhou, China; ^5^Zhejiang Key Laboratory for Research in Assessment of Cognitive Impairments, Hangzhou, China

**Keywords:** COVID-19, childhood trauma, mental health, pandemic, network analysis

## Abstract

**Background:**

Childhood trauma has been found to have an important impact on mental health. However, little is known regarding the intercorrelations between childhood trauma and mental health during the COVID-19 pandemic. This study aimed to investigate such complex interplay between childhood trauma, depression, anxiety, post-traumatic stress level during the COVID-19 pandemic, and fear of COVID-19 using network analysis.

**Methods:**

A total of 1,247 college students were recruited and were asked to complete a series of questionnaires, including the Childhood Trauma Questionnaire, Patient Health Questionnaire, Generalized Anxiety Disorder Scale, Post-traumatic Stress Checklist—Civilian version, and Fear of COVID-19 Scale. The Gaussian graphical model with the scores of the questionnaires as nodes was estimated. The partial correlations between nodes were calculated as edges. Moreover, network comparison tests were conducted to compare the network patterns between participants with high levels of childhood trauma and low levels of childhood trauma.

**Results:**

Childhood trauma was found to be connected to depression, anxiety, and post-traumatic stress level. The node of childhood trauma exhibited the strongest strength and the highest expected influence in the network. Participants with high levels of childhood trauma and participants with low levels of childhood trauma showed comparable network structure and global strength.

**Conclusion:**

Our findings revealed a complex network pattern between childhood trauma and different mental health problems, indicating that childhood trauma might be a risk factor for mental health during the COVID-19 pandemic.

## 1. Introduction

The COVID-19 pandemic has become a global health crisis. Apart from the significant challenges to public health, the pandemic has serious impacts on individual’s mental health worldwide (1). The prevalence of psychological distress, depression, anxiety, and post-traumatic stress symptoms has significantly increased during the pandemic (2–5). Importantly, previous studies suggested that individuals with childhood trauma are at higher risk of experiencing more mental health problems during the COVID-19 pandemic (6, 7).

Childhood trauma refers to experiences that are emotionally or physically harmful or distressing, occurring during the developmental period of childhood (8). Previous studies have documented a relatively high prevalence of childhood trauma in college students (9, 10). For example, a survey among 21 countries by the World Mental Health Initiative recruited 51,945 adults and found approximately 40% of the population had adverse childhood experiences (11). Childhood trauma includes various types, including physical and emotional abuse, sexual abuse, or neglect (12). Different types of childhood trauma have distinct impacts on neuropsychological development (13). Physical and emotional abuse or neglect has been found to lead to emotion dysregulation and cognitive decline (14, 15). It has been considered a risk factor contributing to a wide range of psychological difficulties and mental disorders (16, 17). Moreover, individuals who have been exposed to traumatic events in childhood have been found to have higher levels of fear and stress in response to later stressors (18).

Since the end of 2019, the COVID-19 pandemic has become an ongoing and significant stressor for individuals worldwide (6). Recent studies have explored how individuals with childhood trauma experienced such stressor during the pandemic (7, 19–21). Moreover, significant correlations between adverse childhood experiences and mental health problems during the pandemic have been found (19–21). For example, Guo et al. (19) found that individuals with higher levels of adverse childhood experiences (including physical abuse, emotional abuse, sexual abuse, family neglect, and household dysfunctions) had increased levels of anxiety and post-traumatic stress symptoms. Doom et al. (21) found that higher levels of adverse childhood experiences were significantly associated with higher levels of depression during the COVID-19 pandemic. Furthermore, individuals with childhood abuse have been found to have increased levels of COVID-19 fear and post-traumatic stress symptoms (6). Therefore, previous research indicated that individuals with childhood trauma might be more susceptible to heightened anxiety, depression, and post-traumatic stress symptoms during the COVID-19 pandemic. Previous studies on the mental health consequences of the COVID-19 pandemic have found greater increases in depression, anxiety, and post-traumatic stress level in the general population (2–5, 22, 23). Therefore, the present study will focus on the above three mental health problems.

Network analysis, which is a novel method to investigate the relationships and interactions between different symptoms, has been used extensively in psychology and psychiatry (24, 25). In network analysis, symptoms are represented as nodes, and the relationships between symptoms are represented as edges (26, 27). Importantly, an edge linking two nodes represents the independent relationship after controlling for the effects of the rest nodes in the network. The network structure and the network properties such as centrality and betweenness could be examined. Therefore, network analysis can elucidate the interactive pattern of symptoms (28). Some studies have utilized network analysis to explore the network structure of psychological state during the pandemic (29–32). Feng et al. (29) used a network approach to examine the relationships between depression and intolerance of uncertainty in university students. Ge et al. (30) and Ventura-Leon et al. (32) explored the network structure involving depression and anxiety symptoms. However, limited studies examined the network structure between childhood trauma and other mental health problems.

Therefore, this study aimed to use network analysis to investigate the relationships between childhood trauma, depression, anxiety, post-traumatic stress level during the COVID-19 pandemic, and fear of COVID-19. As previous findings using a non-network approach have found that individuals with higher levels of childhood trauma reported more mental health problems during the COVID-19 pandemic (19–21), we hypothesized that (1) childhood trauma would be connected to mental health problems and fear of COVID-19, (2) the node of childhood trauma would have high centrality indices, and (3) compared with participants with low levels of childhood trauma, participants with high levels of childhood trauma would have more mental health problems. The networks of both groups would exhibit different structures.

## 2. Materials and methods

### 2.1. Participants and procedure

A total of 1,247 students (361 males; mean age = 19.80 years, SD = 1.48) were recruited from Hangzhou Normal University in Hangzhou, China. The participants who gave consent completed a set of self-report questionnaires online. Each participant received 10 RMB as an incentive. The invalid responses were detected using 5 pairs of validity check items (33, 34). Participants with scores over 3 were excluded due to low-quality responses. This study was approved by the Research Ethics Review Board of the School of Basic Medical Sciences, Hangzhou Normal University.

### 2.2. Measures

#### 2.2.1. The childhood trauma questionnaire—short form

The CTS-SF is widely used to assess childhood trauma (35, 36). The CTQ-SF comprises 28 items rated from 1 (never true) to 5 (very often true). The higher scores indicate greater severity of childhood trauma the participant experienced. The CTQ-SF had five subscales: emotional abuse (node: CTQ1), physical abuse (node: CTQ2), sexual abuse (node: CTQ3), emotional neglect (node: CTQ4), and physical neglect (node: CTQ5). The Chinese version of the CTQ-SF has been shown to have good psychometric properties (35). The Cronbach’s alpha for the CTQ-SF in the present study was 0.78.

To compare the network pattern between participants with high and low levels of childhood trauma, the CTS-SF was further used to define participants with high levels of childhood trauma and participants with low levels of childhood trauma. A total CTQ score higher than 1 SD above the mean score of the whole sample was defined as “high levels of childhood trauma” (⩾ 54) and a lower total CTQ score (< 54) was classified as “low levels of childhood trauma” (37–39).

#### 2.2.2. The patient health questionnaire

The PHQ-9 is a 9-item questionnaire for assessing depressive levels (40, 41). Each item is rated on a 5-point Likert scale (from 0 as “no day” to 4 as “almost every day”), with higher scores indicating higher levels of depression. The Chinese version of the PHQ-9 has been found to have good reliability and validity (40). The Cronbach’s alpha for the PHQ-9 in this study was 0.86. The total score of the PHQ-9 was added as an indicator of depressive levels (node: PHQ) in the network analysis.

#### 2.2.3. The generalized anxiety disorder scale

The GAD-7 is a 7-item questionnaire used for measuring generalized anxiety (42, 43). Items of the GAD-7 are rated on a 4-point Likert scale (from 0 as “no day” to 3 as “almost every day”). The higher scores indicate higher levels of anxiety. The Chinese version of the GAD-7 has been found to have good test–retest reliability and convergent validity (42). The Cronbach’s alpha for the GAD-7 in this study was 0.90. The total score of the GAD-7 was added as an indicator of anxiety (node: GAD) in the network analysis.

#### 2.2.4. The post-traumatic stress checklist—civilian version

The PCL-C is a 17-item self-report scale for measuring post-traumatic stress symptoms (44, 45). In the present study, the PCL-C was used to measure the post-traumatic stress level during the COVID-19 pandemic. The PCL-C consists of three subscales, including re-experiencing (node: PCL1), avoidance (node: PCL2), and hyperarousal (node: PCL3). Each item is rated on a 5-point Likert scale (from 1 “not at all” to 5 “extremely bothered”), with higher scores indicating more severe symptoms. The Chinese version of the PCL-C has shown good internal consistency and validity (44). The Cronbach’s alpha for the PCL-C in the present study was 0.93.

#### 2.2.5. The fear of COVID-19 scale

The FCV-19S is a valid tool for assessing individuals’ fear of COVID-19 (46, 47). It contains 7 items with a 5-point Likert scale from 1 (strongly disagree) to 5 (strongly agree). The higher scores of the FCV-19S indicate higher levels of fear of COVID-19. The Chinese version of the FCV-19S has been shown to have good reliability and validity (46). The Cronbach’s alpha for the FCV-19S in the present study was 0.87. The total score of the FCV-19S was added as an indicator of fear of COVID-19 (node: FCV).

### 2.3. Data analyses

#### 2.3.1. Descriptive analyses

All descriptive statistics were computed in IBM SPSS Statistics 21 (IBM Corp., 2012). The mean and standard deviation of age, education duration, and total and subscale scores of all questionnaires were calculated for the entire sample as well as groups of participants with high and low levels of childhood trauma. We examined gender differences in the scores of questionnaires using independent sample *t*-tests. Pearson correlation analysis and partial correlation analysis were conducted to investigate the relationship between the scores of the CTQ-SF and other questionnaires’ scores in the entire sample (Supplementary Table S1). Independent sample *t*-tests were conducted to examine the group differences in fear of COVID-19, depression, anxiety, and post-traumatic level. Moreover, we included age, gender, and years of education as covariates to examine the group differences using analysis of covariance (ANCOVA).

#### 2.3.2. Network estimation

Packages of bootnet (48), qgraph (49), and networktools (50) in R studio software (Version: 2023.06.0 + 421, available at https://posit.co/download/rstudio-desktop/) were used to construct networks in our study. We used the Gaussian graphical model as the estimation model. The partial correlation matrix was computed using the Extended Bayesian Information Criterion Graphical Least Absolute Shrinkage and Selection Operator (EBICglasso) procedure to improve the accuracy and interpretability of our network (51, 52). Bonferroni correction was applied to control for a false positive rate (53). Symptoms were represented as the nodes, and correlations between nodes were represented as the edges, ranging from −1 to 1.

#### 2.3.3. Network centrality

R packages of qgraph (49) and networktools (50) were used to calculate the centrality of nodes in the networks. Centrality was a measure used in a network to quantify the significance of nodes within that network based on the connectedness and interactions of each node with other nodes. Centrality indices, including strength, closeness, and betweenness, were computed. Expected influence is referred to evaluate the predicted significance of certain nodes in the entire network. It was argued that in a network with both positive and negative edges, the expected influence is more appropriate to assess the nature and strength of the significance of nodes (54). Hence, we included the expected influence as the fourth centrality index. Results were converted to *z*-scores to compare different centrality indices on the same scale.

The bridge centrality between clusters of symptoms represented the importance of certain nodes in serving as a connecting link or shared manifestation between various sets of symptoms (55). Subscales of childhood trauma and PTSD were set as two clusters of symptoms. Depression and anxiety were considered a cluster of symptoms. Finally, fear of COVID-19 was set as an independent cluster of the symptoms. Bridge centrality was calculated to identify nodes that connect symptoms of the four different mental disorders.

#### 2.3.4. Network accuracy and stability

R packages of bootnet (Version 1.5) (48) and ggplot2 (56) were used to examine the accuracy and stability of our network. The bootstrapping method was applied with 1,000 iterations. The edge weights accuracy test was conducted to examine the reliability of our network to describe our sample characteristics. The centrality stability test was employed to illustrate the stability of our network across samples, which was quantified by the correlation stability coefficient (CS coefficient). In order to consider a network stable, the coefficient should be at least 0.25 and preferably above 0.5 (48).

#### 2.3.5. Network comparison tests

Network comparison tests (NCTs) were performed using the R package of NetworkComparisonTest to explore the differences between the two networks constructed by samples of participants with high and low levels of childhood trauma as well as between gender-stratified groups (57). A two-tailed permutation test (*n* = 10,000) with *p* < 0.05 was used to compare the global strength, centrality invariance, and edge weights of the two networks. Bonferroni correction was applied in multiple comparisons to control for a false-positive rate (53).

## 3. Results

### 3.1. Sample characteristics

Demographic information and a descriptive summary of the questionnaires’ scores are shown in Table 1. Based on the scores of the CTS-SF, 125 participants had high levels of childhood trauma and the rest of the participants were grouped as having low levels of childhood trauma (*n* = 1,122). The independent sample *t*-test found that participants with high levels of childhood trauma had significantly higher levels of depression, anxiety, fear, and post-traumatic stress symptoms for COVID-19. The results remained unchanged after controlling for age, gender, and years of education (see Supplementary Results and Supplementary Table S2). Male participants had significantly higher levels of childhood trauma and post-traumatic stress symptoms for COVID-19 and lower levels of depression and anxiety than female participants (see Supplementary Table S5).

**Table 1:** Demographic information and descriptive statistics of questionnaire scores.

	Entire sample (*n* = 1,247)	High CTQ (*n* = 125)	Low CTQ (*n* = 1,122)	*t*	*p*	Cohen’s *d*
Demographics						
Age (year)	19.80 (1.48)	19.85 (1.17)	19.79 (1.51)	0.43	0.665	0.04
Edu (year)	13.7 (2.15)	13.68 (1.82)	13.70 (2.19)	−0.08	0.934	0.09
Questionnaire scores						
FCV	17.36 (5.45)	18.92 (6.16)	17.19 (5.34)	3.38	0.001^**^	0.32
PHQ	15.66 (4.26)	18.62 (5.61)	15.33 (3.95)	8.41	0.001^***^	0.79
GAD	12.02 (3.88)	14.22 (4.85)	11.78 (3.68)	6.80	0.001^***^	0.64
PCL						
Total of PCL	30.43 (10.20)	38.34 (12.66)	29.55 (9.50)	9.46	0.001^***^	0.89
PCL1: Re-experiencing	8.91 (3.53)	11.11 (4.27)	8.66 (3.35)	7.52	0.001^***^	0.71
PCL2: Avoidance	12.37 (4.41)	15.54 (5.39)	12.01 (4.14)	8.74	0.001^***^	0.82
PCL3: Hyperarousal	9.15 (3.62)	11.69 (4.34)	8.87 (3.42)	8.50	0.001^***^	0.80
CTQ-SF						
Total of CTQ-SF	45.39 (8.45)	64.82 (11.04)	43.22 (4.34)	42.44	0.001^***^	4.00
CTQ1: Emotional abuse	6.99 (2.58)	11.99 (3.60)	6.43 (1.69)	29.99	0.001^***^	2.83
CTQ2: Physical abuse	5.55 (1.84)	8.82 (4.33)	5.19 (0.59)	26.10	0.001^***^	2.46
CTQ3: Sexual abuse	5.51 (1.86)	8.37 (4.63)	5.19 (0.69)	21.05	0.001^***^	1.98
CTQ4: Emotional neglect	9.45 (3.66)	15.18 (3.50)	8.82 (3.07)	21.61	0.001^***^	2.04
CTQ5: Physical neglect	7.46 (2.69)	11.97 (3.15)	6.95 (2.10)	23.90	0.001^***^	2.27

High CTQ, participants with high levels of childhood trauma; Low CTQ, participants with low levels of childhood trauma; Edu, education duration; CV, fear of Coronavirus disease 2019 (COVID-19) scale; PHQ, patient health questionnaire; GAD, generalized anxiety disorder questionnaire; PCL, post-traumatic stress disorder checklist—civilian; Total, total score of the post-traumatic stress disorder checklist—civilian; PCL1, re-experiencing; PCL2, avoidance; PCL3, hyperarousal; CTQ, childhood trauma questionnaire; CTQ1, emotional abuse; CTQ2, physical abuse; CTQ3, sexual abuse; CTQ4, emotional neglect; CTQ5, physical neglect.

### 3.2. Network estimation

**Figure 1:**
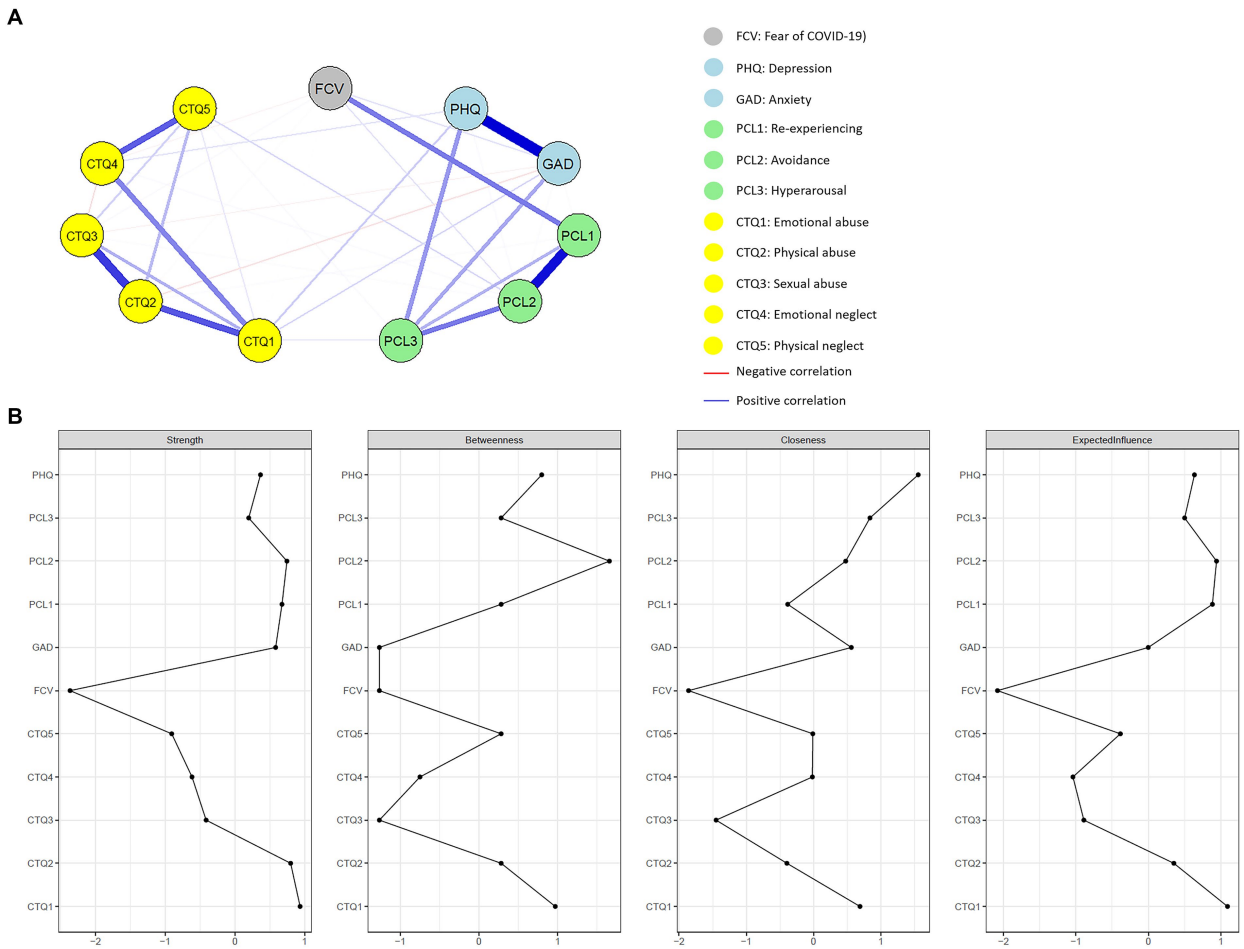
**(A)** Visualization of the regularized network for the entire sample. Clusters of symptoms are represented by different colors. The gray node represents the fear of COVID-19. The light blue nodes represent depression and anxiety. The green nodes are a combination of traumatic symptoms post-COVID-19. Symptoms of childhood trauma are represented by the yellow nodes. Blue lines mean positive connections between nodes, whereas red lines mean negative connections. Thicker and shorter edges represent larger partial correlations. **(B)** Centrality indices plot include strength, betweenness, closeness, and expected influence. Values were converted to standardized *z*-scores. FCV, fear of Coronavirus disease 2019 (COVID-19) scale; PHQ, patient health questionnaire; GAD, generalized anxiety disorder questionnaire; PCL, post-traumatic stress disorder checklist—civilian; CTQ, childhood trauma questionnaire.

The network visualization of the entire sample (*n* = 1,247) with 11 nodes is shown in Figure 1. As expected, positive correlations were found between the nodes that represented the subscales, indicating relatively strong “within-subscale” edges. As for the “between-scale” edges, the strongest one was the edge between the PHQ (the score of depression) and the GAD (the score of anxiety) (regularized partial correlation = 0.57), followed by the edge between the FCV (the score of fear of COVID-19) and the PCL1 (the score of the re-experiencing subscale) (regularized partial correlation = 0.29). Moreover, the PCL3 (the score of the hyperarousal subscale) was positively connected to the PHQ (the score of depression) and the GAD (the score of anxiety) (the partial correlations were 0.22 and 0.19, respectively). The CTQ1 (the score of emotional abuse subscale) was found to be positively connected to the PHQ (the score of depression), the GAD (the score of anxiety), and the PCL2 (the score of the avoidance subscale), and the CTQ5 (the score of the physical neglect subscale) was found to be positively connected to the PCL3 (the score of the hyperarousal subscale).

### 3.3. Network centrality

Figure 1 and Table 2 display the four centrality indices of the 11 nodes, which reflect the structural position and significance of each node. The CTQ1 (the score of emotional abuse subscale), the CTQ2 (the score of the physical abuse subscale), and the PCL2 (the score of the avoidance subscale) were the top three nodes in strength centrality, indicating that these nodes had the strongest connections with other nodes in the network. As for the betweenness centrality, the PCL2 (the score of avoidance subscale), the CTQ1 (the score of emotional abuse subscale), and the PHQ (the score of depression) had the most interactions with other nodes in the network. Regarding the closeness centrality, the PHQ (the score of depression), the PCL3 (the score of the hyperarousal subscale), and the CTQ1 (the score of emotional abuse subscale) had the shortest average distance with the other nodes, which suggested that these nodes were more closely connected with the other nodes in the network.

**Table 2:** Centrality and expected influence of nodes in the network.

	Strength	Closeness	Betweenness	Expected influence
FCV	−2.365	−1.859	−1.266	−2.083
PHQ	0.366	1.555	0.797	0.636
GAD	0.584	0.561	−1.266	−0.002
PCL				
PCL1: Re-experiencing	0.675	−0.385	0.281	0.884
PCL2: Avoidance	0.746	0.477	1.656	0.941
PCL3: Hyperarousal	0.197	0.838	0.281	0.500
CTQ				
CTQ1: Emotional abuse	0.934	0.689	0.969	1.092
CTQ2: Physical abuse	0.799	−0.397	0.281	0.351
CTQ3: Sexual abuse	−0.413	−1.450	−1.266	−0.891
CTQ4: Emotional neglect	−0.616	−0.018	−0.750	−1.041
CTQ5: Physical neglect	−0.907	−0.012	0.281	−0.387

FCV, fear of Coronavirus disease 2019 (COVID-19) scale; PHQ, patient health questionnaire; GAD, generalized anxiety disorder questionnaire; PCL, post-traumatic stress disorder checklist—civilian; CTQ, childhood trauma questionnaire.

**Figure 2:**
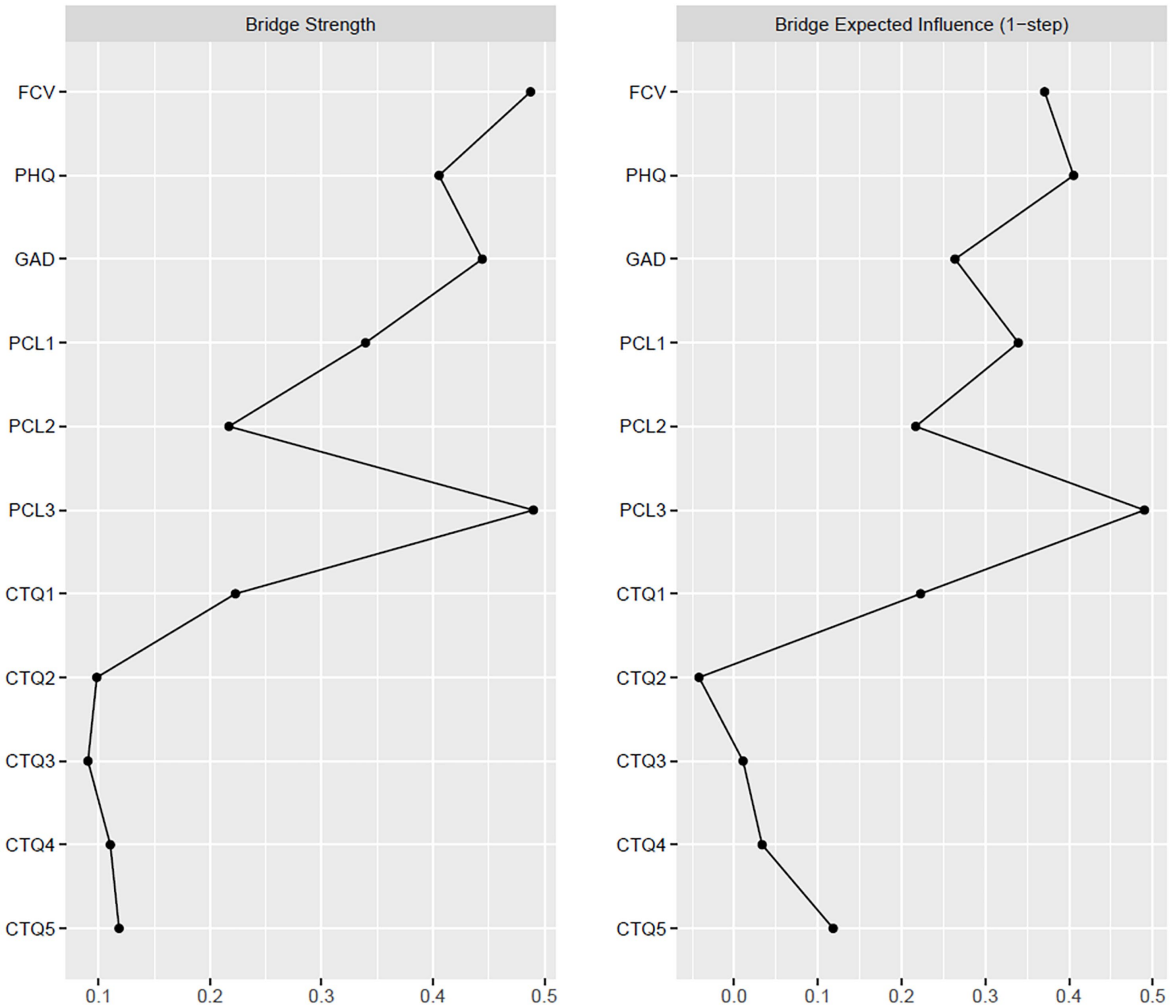
Bridge centrality indices plots of strength (left) and expected influence (right) for the entire sample (*n* = 1,247). FCV, fear of Coronavirus disease 2019 (COVID-19) scale; PHQ, patient health questionnaire; GAD, generalized anxiety disorder questionnaire; PCL, post-traumatic stress disorder checklist—civilian; PCL1, re-experiencing; PCL2, avoidance; PCL3, hyperarousal; CTQ, childhood trauma questionnaire; CTQ1, emotional abuse; CTQ2, physical abuse; CTQ3, sexual abuse; CTQ4, emotional neglect; CTQ5, physical neglect.

The results of the bridge centrality test are shown in Figure 2 and Supplementary Table S3. The PCL3 (the score of the hyperarousal subscale) had the greatest centrality in both the strength and expected influence, showing the occurrence of hyperarousal in all of the four clusters of symptoms.

### 3.4. Network accuracy and stability

**Figure 3:**
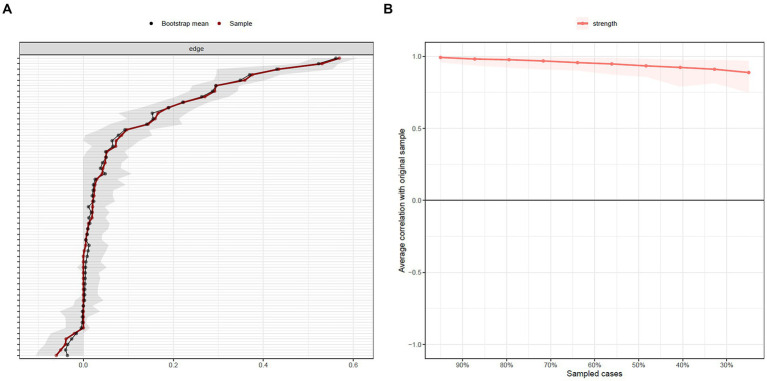
**(A)** Bootstrapped confidence intervals (CIs) of edge weights for the entire sample (*n* = 1,247). Dots fitted on the red line are sample edge weights and on the black line are bootstrapped means. The gray area indicates the 95% CIs. **(B)** Average correlations of strength centrality with the original sample when *n* % of sample cases were dropped. Lines indicate the means and the areas indicate the range from the 2.5 quantile to the 97.5 quantile.

Figure 3 presents the network stability and accuracy test of the entire sample. Results showed a strength centrality stability coefficient of 0.75, meaning excellent stability of the network. Additionally, as illustrated in Figure 3, the accuracy test showed small confidence intervals (CIs), indicating a reliable estimation of the edge weights.

### 3.5. Network comparison test

**Figure 4:**
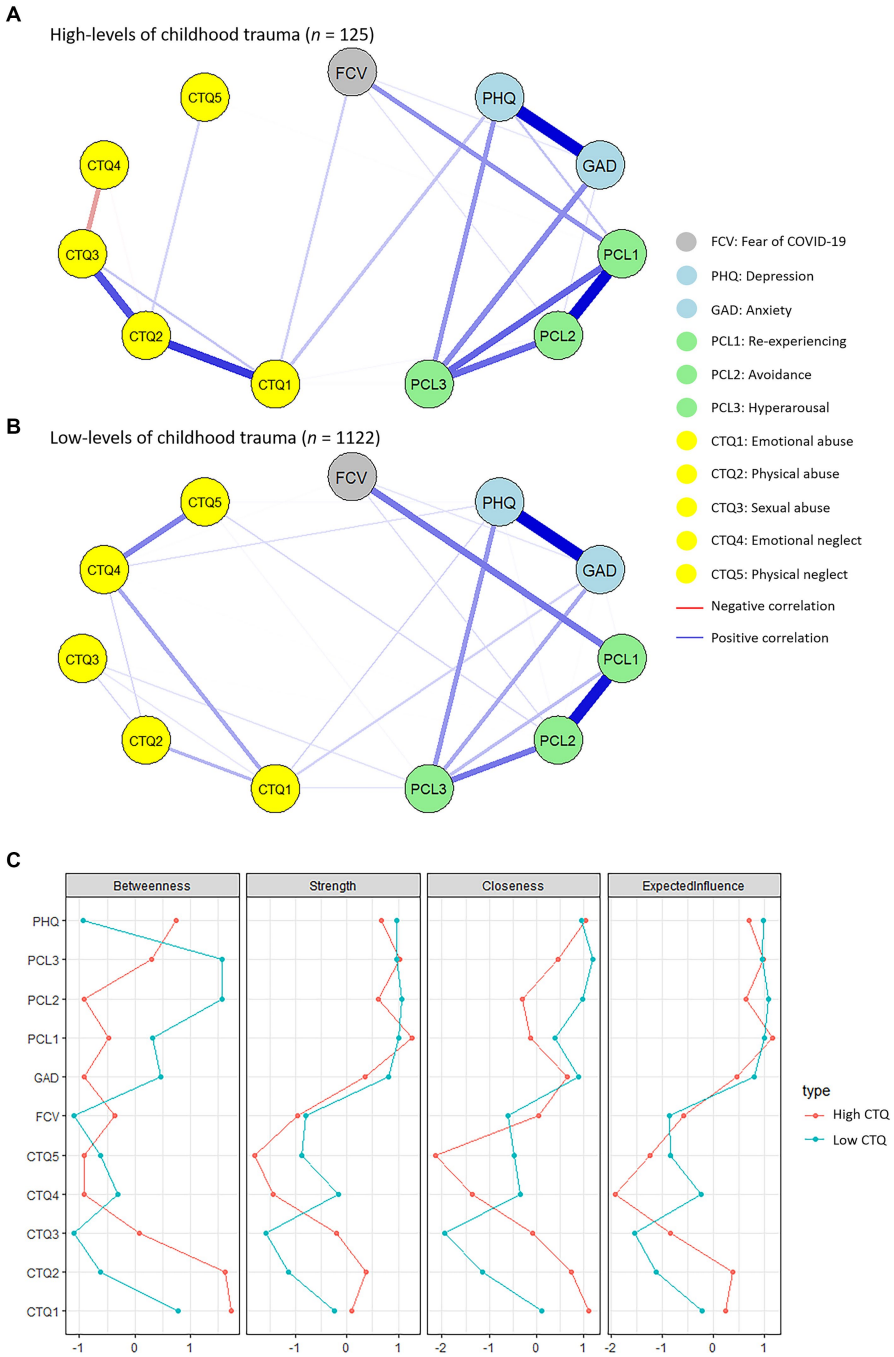
**(A)** Network visualization of people with high levels of childhood trauma (*n* = 125). **(B)** Network visualization of people with low levels of childhood trauma (*n* = 1,122). The gray nodes represent the fear of COVID-19. The light blue nodes represent depression and anxiety. The green nodes are a combination of traumatic symptoms post-COVID-19. Symptoms of childhood trauma are represented by the yellow nodes. Blue lines are positive correlations between nodes, and red lines are negative correlations. The thicker and shorter the lines, the stronger and closer the connections. FCV, fear of Coronavirus disease 2019 (COVID-19) scale; PHQ, patient health questionnaire; GAD, generalized anxiety disorder questionnaire; PCL, post-traumatic stress disorder checklist—civilian; CTQ, childhood trauma questionnaire. **(C)** Centrality indices plot, including strength, betweenness, closeness, and expected influence, of the two networks. The red line represents individuals with high levels of childhood trauma and the blue line represents individuals with low levels of childhood trauma. Centrality values are presented in *z*-scores.

The visualized networks of the sample with high (Figure 4A; *n* = 125) and low (Figure 4B; *n* = 1,122) levels of childhood trauma as well as their centrality test are presented in Figure 4. The centrality and expected influence of the network for the two groups are shown in Supplementary Table S4. The global strength invariance test found no significant differences between the two networks (the strength difference was 0.28, *p* = 0.645), indicating that the two networks shared similar global strength of connections. The network invariance test also found no significant differences between the two networks (*M* = 0.27, *p* = 0.309). However, the node with the strongest betweenness for participants with high levels of childhood trauma was the CTQ1 (the score of the emotional abuse subscale), while the PCL3 (the score of the hyperarousal subscale) was the node with the strongest betweenness in the network for participants with low levels of childhood trauma. Moreover, the edge invariance test shows significant differences in FCV-CTQ1, CTQ3-CTQ4, and CTQ4-CTQ5. In addition, the network comparison test did not find a significant difference between the male and the female groups (Supplementary Table S6).

## 4. Discussion

Applying network analysis, the network structure between various types of childhood trauma and mental health problems during the COVID-19 pandemic was modeled. Consistent with our hypothesis, we found that the nodes of childhood trauma were connected to depression, anxiety, and post-traumatic stress level. Moreover, the nodes of childhood trauma showed the strongest strength centrality, indicating the important role of childhood trauma in the network. The network comparison test between participants with high and low levels of childhood trauma revealed different network structures. Furthermore, compared with participants with low levels of childhood trauma, participants with high levels of childhood trauma showed significantly higher levels of depression, anxiety, fear, and post-traumatic stress for COVID-19. Our findings suggest that individuals who have experienced childhood trauma may be more susceptible to experiencing these mental health difficulties during the COVID-19 pandemic.

Childhood trauma (the CTQ1 node and the CTQ2 node) was found to have the strongest strength centrality in the network based on the whole sample, indicating that childhood trauma played a central role in the network structure and highlighting the significant impact that childhood trauma can have on mental health outcomes during the pandemic. Our findings are consistent with previous studies which found positive correlations between childhood trauma and mental health problems during the COVID-19 pandemic (6, 58). Regarding the betweenness centrality and the closeness centrality, the findings suggested that the post-traumatic stress level (the PCL2 node and the PCL3 node), depression (the PHQ node), and childhood trauma (the CTQ1 node) played an important role in the network. These findings are in line with previous findings (59, 60). Childhood trauma we measured includes emotional abuse (the CTQ1 node), physical abuse (the CTQ2 node), sexual abuse (the CTQ3 node), emotional neglect (the CTQ4 node), and physical neglect (the CTQ5 node). Our findings found that emotional abuse has the highest centrality indexed in the whole network. Notably, individuals with more childhood trauma (especially emotional abuse) have been found to experience more psychological distress during the coronavirus (61). Therefore, interventions on emotion regulation mechanisms (62) may have potential effects on mental health for individuals with childhood trauma during the COVID-19 pandemic. Although studies using network analysis to examine the impacts of childhood trauma on mental health were scarce, some authors have adopted this approach to explore the impacts of the COVID-19 pandemic on mental health (63–66). For example, Zavlis et al. (63) included depression, anxiety, trauma symptoms, COVID-specific anxiety, and viral exposure to construct the network. They found no associations between viral exposure and symptoms, which may support our findings of weak connections between the FCV node with other nodes (63). Furthermore, Ventura-Leon et al. (64) revealed that the symptoms of depression and anxiety were the most central symptoms in their network. Meanwhile, they found that the depressive symptoms bridged the symptoms of stress and anxiety (64), which was similar to our network structure. Bridge centrality is usually used to understand comorbidity in network analysis (55). Hyperarousal had the highest bridge centrality in our study, demonstrating its significance in the development of both fear of the pandemic, depression, anxiety, and PTSD problems and its high co-occurrence with childhood maltreatment. This result was in line with the study, which found that hyperarousal symptoms have a mediation effect between childhood maltreatment and depression (67). Our findings indicate that hyperarousal might be a risk factor for mental health problems.

Additionally, when comparing the network structures between participants with high and low levels of childhood trauma, different structure was observed. This suggests that the presence and severity of childhood trauma can lead to variations in the network connections and dynamics of mental health problems during the pandemic. In terms of group effect, participants with high levels of childhood trauma displayed significantly more mental health problems compared with those with low levels of childhood trauma. Such findings are consistent with previous findings, which demonstrated more trauma symptoms and mental distress in individuals with childhood abuse (60, 68). Moreover, it has been widely recognized that childhood trauma is a risk factor contributing to the development of mental disorders, such as affective disorders, post-traumatic stress disorder, and schizophrenia (15, 69, 70).

It is important to note that this study has several limitations. First, the data collected relied on self-report measures, which may be subject to recall bias or social desirability bias. Second, the participants recruited in our study were college students. The findings may not be generalizable to other populations. Future research should aim to replicate these findings in diverse samples. Finally, the sample size of the group with high levels of childhood trauma was relatively small. A larger sample size is recommended for future studies to conduct network comparison tests.

In conclusion, the present study applied network analysis to investigate the intercorrelations of childhood trauma, depression, anxiety, fear, and post-traumatic stress levels related to COVID-19. The findings support the specific links between childhood trauma and mental health problems. These findings underscore the critical need for targeted interventions and support for individuals who have experienced childhood trauma, particularly during times of the COVID-19 pandemic.

## Data availability statement

The raw data supporting the conclusions of this article will be made available by the authors, without undue reservation.

## Ethics statement

The studies involving humans were approved by the Research Ethics Review Board of the School of Basic Medical Sciences, Hangzhou Normal University. The studies were conducted in accordance with the local legislation and institutional requirements. The participants provided their written informed consent to participate in this study.

## Author contributions

X-lC and HHei collaboratively designed the study. JX and HHua recruited participants and collected data. LZ analyzed the data. X-lC, LZ, and JX wrote the drafts of the manuscript. PF, MZ, and HHei interpreted the findings and commented on the manuscript critically. All authors contributed to the article and approved the submitted version.

## Funding

This study was supported by a grant from the Research Project of Shanghai Science and Technology Commission (20dz2260300), the Fundamental Research Funds for the Central Universities, and the Starting Research Fund from Hangzhou Normal University.

## Conflict of interest

The authors declare that the research was conducted in the absence of any commercial or financial relationships that could be construed as a potential conflict of interest.

## Publisher’s note

All claims expressed in this article are solely those of the authors and do not necessarily represent those of their affiliated organizations, or those of the publisher, the editors and the reviewers. Any product that may be evaluated in this article, or claim that may be made by its manufacturer, is not guaranteed or endorsed by the publisher.

## References

[1] Salari N Hosseinian-Far A Jalali R Vaisi-Raygani A Rasoulpoor S Mohammadi M. Prevalence of stress, anxiety, depression among the general population during the COVID-19 pandemic: a systematic review and meta-analysis. Glob Health. (2020) 16:57. doi: 10.1186/s12992-020-00589-w, PMID: 32631403 PMC7338126

[2] Liu Y Zou L Yan S Zhang P Zhang J Wen J. Burnout and post-traumatic stress disorder symptoms among medical staff two years after the COVID-19 pandemic in Wuhan, China: social support and resilience as mediators. J Affect Disord. (2023) 321:126–33. doi: 10.1016/j.jad.2022.10.027, PMID: 36280200 PMC9585849

[3] Daly M Robinson E. Acute and longer-term psychological distress associated with testing positive for COVID-19: longitudinal evidence from a population-based study of US adults. Psychol Med. (2023) 53:1603–10. doi: 10.1017/S003329172100324X 34308807 PMC8353189

[4] Fang X Zhang J Teng C Zhao K Su KP Wang Z. Depressive symptoms in the front-line non-medical workers during the COVID-19 outbreak in Wuhan. J Affect Disord. (2020) 276:441–5. doi: 10.1016/j.jad.2020.06.078, PMID: 32871675 PMC7365080

[5] Castro-de-Araujo LFS Rodrigues EDS Machado DB Henriques CMP Verotti MP Goncalves AQ. Multimorbidity worsened anxiety and depression symptoms during the COVID-19 pandemic in Brazil. J Affect Disord. (2022) 314:86–93. doi: 10.1016/j.jad.2022.07.005, PMID: 35810830 PMC9259509

[6] Tsur N Abu-Raiya H. COVID-19-related fear and stress among individuals who experienced child abuse: the mediating effect of complex posttraumatic stress disorder. Child Abuse Negl. (2020) 110:104694. doi: 10.1016/j.chiabu.2020.104694, PMID: 32900515 PMC7430290

[7] Gewirtz-Meydan A Lassri D. A profile analysis of COVID-19 stress-related reactions: the importance of early childhood abuse, psychopathology, and interpersonal relationships. Child Abuse Negl. (2022) 130:105442. doi: 10.1016/j.chiabu.2021.105442, PMID: 34920898 PMC8666322

[8] Morgan C Fisher H. Environment and schizophrenia: environmental factors in schizophrenia: childhood trauma--a critical review. Schizophr Bull. (2007) 33:3–10. doi: 10.1093/schbul/sbl053, PMID: 17105965 PMC2632300

[9] Read JP Ouimette P White J Colder C Farrow S. Rates of DSM-IV-TR trauma exposure and posttraumatic stress disorder among newly matriculated college students. Psychol Trauma. (2011) 3:148–56. doi: 10.1037/a0021260, PMID: 25621098 PMC4301258

[10] Strand EB Brandt J Rogers K Fonken L Chun R Conlon P. Adverse childhood experiences among veterinary medical students: a multi-site study. J Vet Med Educ. (2017) 44:260–7. doi: 10.3138/jvme.0816-123R, PMID: 28346049

[11] Kessler RC McLaughlin KA Green JG Gruber MJ Sampson NA Zaslavsky AM. Childhood adversities and adult psychopathology in the WHO world mental health surveys. Br J Psychiatry. (2010) 197:378–85. doi: 10.1192/bjp.bp.110.080499, PMID: 21037215 PMC2966503

[12] Miller AB Esposito-Smythers C Weismoore JT Renshaw KD. The relation between child maltreatment and adolescent suicidal behavior: a systematic review and critical examination of the literature. Clin Child Fam Psychol Rev. (2013) 16:146–72. doi: 10.1007/s10567-013-0131-5, PMID: 23568617 PMC3724419

[13] McLaughlin KA Sheridan MA Lambert HK. Childhood adversity and neural development: deprivation and threat as distinct dimensions of early experience. Neurosci Biobehav Rev. (2014) 47:578–91. doi: 10.1016/j.neubiorev.2014.10.012, PMID: 25454359 PMC4308474

[14] Hackman DA Farah MJ. Socioeconomic status and the developing brain. Trends Cogn Sci. (2009) 13:65–73. doi: 10.1016/j.tics.2008.11.003, PMID: 19135405 PMC3575682

[15] Aas M Dazzan P Fisher HL Morgan C Morgan K Reichenberg A. Childhood trauma and cognitive function in first-episode affective and non-affective psychosis. Schizophr Res. (2011) 129:12–9. doi: 10.1016/j.schres.2011.03.017, PMID: 21601792

[16] van Nierop M Viechtbauer W Gunther N van Zelst C de Graaf R Ten Have M. Childhood trauma is associated with a specific admixture of affective, anxiety, and psychosis symptoms cutting across traditional diagnostic boundaries. Psychol Med. (2015) 45:1277–88. doi: 10.1017/S0033291714002372, PMID: 25273550

[17] Pruessner M Cullen AE Aas M Walker EF. The neural diathesis-stress model of schizophrenia revisited: an update on recent findings considering illness stage and neurobiological and methodological complexities. Neurosci Biobehav Rev. (2017) 73:191–218. doi: 10.1016/j.neubiorev.2016.12.013, PMID: 27993603

[18] Dykshoorn KL. Trauma-related obsessive-compulsive disorder: a review. Health Psychol Behav Med. (2014) 2:517–28. doi: 10.1080/21642850.2014.905207, PMID: 25750799 PMC4346088

[19] Guo J Fu M Liu D Zhang B Wang X van IJzendoorn MH. Is the psychological impact of exposure to COVID-19 stronger in adolescents with pre-pandemic maltreatment experiences? A survey of rural Chinese adolescents. Child Abuse Negl. (2020) 110:104667. doi: 10.1016/j.chiabu.2020.104667, PMID: 32859393 PMC7440157

[20] De Rubeis V Gonzalez A de Groh M Jiang Y Erbas Oz U Tarride JE. Obesity and adverse childhood experiences in relation to stress during the COVID-19 pandemic: an analysis of the Canadian longitudinal study on aging. Int J Obes. (2023) 47:197–206. doi: 10.1038/s41366-023-01258-9 PMC9868513 36690842

[21] Doom JR Seok D Narayan AJ Fox KR. Adverse and benevolent childhood experiences predict mental health during the COVID-19 pandemic. Advers Resil Sci. (2021) 2:193–204. doi: 10.1007/s42844-021-00038-6, PMID: 33907733 PMC8062213

[22] Kim AW Nyengerai T Mendenhall E. Evaluating the mental health impacts of the COVID-19 pandemic: perceived risk of COVID-19 infection and childhood trauma predict adult depressive symptoms in urban South Africa. Psychol Med. (2022) 52:1587–99. doi: 10.1017/S0033291720003414, PMID: 32895082 PMC7520640

[23] Jung SJ Jun JY. Mental health and psychological intervention amid COVID-19 outbreak: perspectives from South Korea. Yonsei Med J. (2020) 61:271–2. doi: 10.3349/ymj.2020.61.4.271, PMID: 32233168 PMC7105405

[24] Borsboom D Cramer AO. Network analysis: an integrative approach to the structure of psychopathology. Annu Rev Clin Psychol. (2013) 9:91–121. doi: 10.1146/annurev-clinpsy-050212-185608 23537483

[25] Fried EI van Borkulo CD Cramer AO Boschloo L Schoevers RA Borsboom D. Mental disorders as networks of problems: a review of recent insights. Soc Psychiatry Psychiatr Epidemiol. (2017) 52:1–10. doi: 10.1007/s00127-016-1319-z, PMID: 27921134 PMC5226976

[26] Robinaugh DJ Hoekstra RHA Toner ER Borsboom D. The network approach to psychopathology: a review of the literature 2008–2018 and an agenda for future research. Psychol Med. (2020) 50:353–66. doi: 10.1017/S0033291719003404, PMID: 31875792 PMC7334828

[27] Borsboom D. A network theory of mental disorders. World Psychiatry. (2017) 16:5–13. doi: 10.1002/wps.20375, PMID: 28127906 PMC5269502

[28] McNally RJ. Network analysis of psychopathology: controversies and challenges. Annu Rev Clin Psychol. (2021) 17:31–53. doi: 10.1146/annurev-clinpsy-081219-092850, PMID: 33228401

[29] Feng TW Ren L Liu C Li KL Wu L Wei XY. The relations between different components of intolerance of uncertainty and symptoms of depression during the COVID-19 pandemic: a network analysis. Front Psych. (2022) 13:993814. doi: 10.3389/fpsyt.2022.993814 PMC9613443 36311506

[30] Ge FF Zheng AN Wan MT Luo G Zhang J. Psychological state among the general Chinese population before and during the COVID-19 epidemic: a network analysis. Front Psych. (2021) 12:12. doi: 10.3389/fpsyt.2021.591656 PMC7952988 33716811

[31] Jiang WY Ren ZH Yu LX Tan YF Shi CR. A network analysis of post-traumatic stress disorder symptoms and correlates during the COVID-19 pandemic. Front Psych. (2020) 11:568037. doi: 10.3389/fpsyt.2020.568037 PMC7683419 33240124

[32] Ventura-Leon J Caycho-Rodriguez T Talledo-Sanchez K Casiano-Valdivieso K. Depression, COVID-19 anxiety, subjective well-being, and academic performance in university students with COVID-19-infected relatives: a network analysis. Front Psychol. (2022) 13:13. doi: 10.3389/fpsyg.2022.837606 PMC8867004 35222215

[33] Yan YJ Hu HX Wang LL Zhang YJ Lui SSY Huang J. Negative schizotypal traits predict the reduction of reward motivation in effort-reward imbalance. Eur Arch Psychiatry Clin Neurosci. (2022) 273:439–45. doi: 10.1007/s00406-022-01419-3 35637380

[34] Wang K Yu XY Yu CR Liu YF Chu MY Zhang RT. Validation of the Chinese version of the body image concern inventory. Eval Health Prof. (2022) 45:204–14. doi: 10.1177/0163278720979651, PMID: 33322941

[35] He J Zhong X Gao Y Xiong G Yao S. Psychometric properties of the Chinese version of the childhood trauma questionnaire-short form (CTQ-SF) among undergraduates and depressive patients. Child Abuse Negl. (2019) 91:102–8. doi: 10.1016/j.chiabu.2019.03.009 30856597

[36] Bernstein DP Stein JA Newcomb MD Walker E Pogge D Ahluvalia T. Development and validation of a brief screening version of the childhood trauma questionnaire. Child Abuse Negl. (2003) 27:169–90. doi: 10.1016/S0145-2134(02)00541-0, PMID: 12615092

[37] Roy A Gorodetsky E Yuan Q Goldman D Enoch MA. Interaction of FKBP5, a stress-related gene, with childhood trauma increases the risk for attempting suicide. Neuropsychopharmacology. (2010) 35:1674–83. doi: 10.1038/npp.2009.236, PMID: 20090668 PMC2962602

[38] Enoch MA Hodgkinson CA Yuan Q Shen PH Goldman D Roy A. The influence of GABRA2, childhood trauma, and their interaction on alcohol, heroin, and cocaine dependence. Biol Psychiatry. (2010) 67:20–7. doi: 10.1016/j.biopsych.2009.08.019, PMID: 19833324 PMC2964936

[39] Roy A Hodgkinson CA Deluca V Goldman D Enoch MA. Two HPA axis genes, CRHBP and FKBP5, interact with childhood trauma to increase the risk for suicidal behavior. J Psychiatr Res. (2012) 46:72–9. doi: 10.1016/j.jpsychires.2011.09.009, PMID: 21978546 PMC3506169

[40] Wang W Bian Q Zhao Y Li X Wang W Du J. Reliability and validity of the Chinese version of the patient health questionnaire (PHQ-9) in the general population. Gen Hosp Psychiatry. (2014) 36:539–44. doi: 10.1016/j.genhosppsych.2014.05.021, PMID: 25023953

[41] Kroenke K Spitzer RL Williams JB. The PHQ-9: validity of a brief depression severity measure. J Gen Intern Med. (2001) 16:606–13. doi: 10.1046/j.1525-1497.2001.016009606.x, PMID: 11556941 PMC1495268

[42] He X Li C Qian J Cui H Wu W. Reliability and validity of a generalized anxiety disorder scale in general hospital outpatients. Shanghai Arch Psychiatry. (2010) 22:200–3.

[43] Spitzer RL Kroenke K Williams JB Lowe B. A brief measure for assessing generalized anxiety disorder: the GAD-7. Arch Intern Med. (2006) 166:1092–7. doi: 10.1001/archinte.166.10.1092 16717171

[44] Yang X Yang H Liu Q Yang L. The research on the reliability and validity of PCL-C and influence factors. Chin J Health Psychol. (2007) 15:6–9.

[45] Weathers FW Litz BT Herman DS Huska JA Keane TM. (eds.). The PTSD checklist (PCL): reliability, validity, and diagnostic utility. In annual convention of the international society for traumatic stress studies. *Vol. 462*. San Antonio, TX (1993).

[46] Chi X Chen S Chen Y Chen D Yu Q Guo T. Psychometric evaluation of the fear of COVID-19 scale among Chinese population. Int J Ment Health Addict. (2022) 20:1273–88. doi: 10.1007/s11469-020-00441-7, PMID: 33456407 PMC7799163

[47] Ahorsu DK Lin CY Imani V Saffari M Griffiths MD Pakpour AH. The fear of COVID-19 scale: development and initial validation. Int J Ment Health Addict. (2022) 20:1537–45. doi: 10.1007/s11469-020-00270-8, PMID: 32226353 PMC7100496

[48] Epskamp S Borsboom D Fried EI. Estimating psychological networks and their accuracy: a tutorial paper. Behav Res Methods. (2018) 50:195–212. doi: 10.3758/s13428-017-0862-1, PMID: 28342071 PMC5809547

[49] Epskamp S Cramer AO Waldorp LJ Schmittmann VD Borsboom D. Qgraph: network visualizations of relationships in psychometric data. J Stat Softw. (2012) 48:1–18. doi: 10.18637/jss.v048.i04

[50] Jones PJ. Networktools: tools for identifying important nodes in networks. *R package version* (2018) 1:10–1155.

[51] Foygel R Drton M. Extended Bayesian information criteria for Gaussian graphical models. Adv Neural Inf Proces Syst. (2010) 23:604–12.

[52] Chen J Chen Z. Extended Bayesian information criteria for model selection with large model spaces. Biometrika. (2008) 95:759–71. doi: 10.1093/biomet/asn034

[53] Armstrong RA. When to use the Bonferroni correction. Ophthalmic Physiol Opt. (2014) 34:502–8. doi: 10.1111/opo.12131 24697967

[54] Robinaugh DJ Millner AJ McNally RJ. Identifying highly influential nodes in the complicated grief network. J Abnorm Psychol. (2016) 125:747–57. doi: 10.1037/abn0000181, PMID: 27505622 PMC5060093

[55] Jones PJ Ma R McNally RJ. Bridge centrality: a network approach to understanding comorbidity. Multivariate Behav Res. (2021) 56:353–67. doi: 10.1080/00273171.2019.1614898, PMID: 31179765

[56] Wickham H. Chang W Wickham MH. (2016). Package ‘ggplot2’. Create elegant data visualisations using the grammar of graphics. *Version*. 2:1–189.

[57] van Borkulo CD van Bork R Boschloo L Kossakowski JJ Tio P Schoevers RA. Comparing network structures on three aspects: a permutation test. Psychol Methods. (2022). doi: 10.1037/met0000476 35404628

[58] Zhu Z Li P Hao L. Correlation of childhood psychological abuse and neglect with mental health in Chinese college students during the COVID-19 pandemic. Front Psych. (2021) 12:770201. doi: 10.3389/fpsyt.2021.770201 PMC8766813 35069277

[59] Zhao YJ Zhang C Guo T Sha S Su Z Cheung T. Associations between post-traumatic stress symptoms and quality of life among psychiatric healthcare personnel in China during the COVID-19 pandemic: a network approach. Front Psych. (2023) 14:975443. doi: 10.3389/fpsyt.2023.975443, PMID: 36873200 PMC9975756

[60] Asmundson GJ Katz J. Understanding the co-occurrence of anxiety disorders and chronic pain: state-of-the-art. Depress Anxiety. (2009) 26:888–901. doi: 10.1002/da.20600 19691031

[61] Janiri D Moccia L Dattoli L Pepe M Molinaro M De Martin V. Emotional dysregulation mediates the impact of childhood trauma on psychological distress: first Italian data during the early phase of COVID-19 outbreak. Aust N Z J Psychiatry. (2021) 55:1071–8. doi: 10.1177/0004867421998802, PMID: 33715469

[62] Fava GA Cosci F Sonino N. Current psychosomatic practice. Psychother Psychosom. (2017) 86:13–30. doi: 10.1159/000448856 27884006

[63] Zavlis O Butter S Bennett K Hartman TK Hyland P Mason L. How does the COVID-19 pandemic impact on population mental health? A network analysis of COVID influences on depression, anxiety and traumatic stress in the UK population. Psychol Med. (2021) 52:3825–33. doi: 10.1017/S0033291721000635 PMC8010290 33722329

[64] Ventura-Leon J Lopez-Jurado R Porturas E Leon-Mostacero I Canchanya-Balbin SE. Anxiety, depression, stress, worry about COVID-19 and fear of loneliness during COVID-19 lockdown in Peru: a network analysis approach. Front Public Health. (2022) 10:946697. doi: 10.3389/fpubh.2022.946697, PMID: 36159279 PMC9500506

[65] Cha EJ Jeon HJ Chung S. Central symptoms of insomnia in relation to depression and COVID-19 anxiety in general population: a network analysis. J Clin Med. (2022) 11:3416. doi: 10.3390/jcm11123416, PMID: 35743484 PMC9224757

[66] Jin Y Sha S Tian T Wang Q Liang S Wang Z. Network analysis of comorbid depression and anxiety and their associations with quality of life among clinicians in public hospitals during the late stage of the COVID-19 pandemic in China. J Affect Disord. (2022) 314:193–200. doi: 10.1016/j.jad.2022.06.051, PMID: 35780965 PMC9242942

[67] Kwon A Lee HS Lee SH. The mediation effect of hyperarousal symptoms on the relationship between childhood physical abuse and suicidal ideation of patients with PTSD. Front Psych. (2021) 12:613735. doi: 10.3389/fpsyt.2021.613735, PMID: 33841200 PMC8032896

[68] Tsur N Shahar G Defrin R Lahav Y Ginzburg K. Torturing personification of chronic pain among torture survivors. J Psychosom Res. (2017) 99:155–61. doi: 10.1016/j.jpsychores.2017.06.016, PMID: 28712422

[69] Yehuda R Halligan SL Grossman R. Childhood trauma and risk for PTSD: relationship to intergenerational effects of trauma, parental PTSD, and cortisol excretion. Dev Psychopathol. (2001) 13:733–53. doi: 10.1017/S0954579401003170, PMID: 11523857

[70] Heim C Newport DJ Mletzko T Miller AH Nemeroff CB. The link between childhood trauma and depression: insights from HPA axis studies in humans. Psychoneuroendocrinology. (2008) 33:693–710. doi: 10.1016/j.psyneuen.2008.03.008, PMID: 18602762

