# Supplementary material for: Relationships between childhood trauma and mental health during the COVID-19 pandemic: a network analysis
Source: Front Psychiatry. 2023 Sep 8;14:1251473. doi: 10.3389/fpsyt.2023.1251473 (PMC10515217; doi:10.3389/fpsyt.2023.1251473)
Supplement: Supplementary file 1 [file Data_Sheet_1.docx]

**Relationships between childhood trauma and mental health during the COVID-19 Pandemic: a network analysis**

**Supplementary materials**

1. **Supplementary Methods**
2. **Supplementary Results**
3. **Supplementary Table S1.** Pearson’s correlations between childhood trauma and other variates.
4. **Supplementary Table S2.** ANCOVA analysis between high- and low-levels of childhood trauma after taking age, education duration, and gender into consideration.
5. **Supplementary Table S3.** Bridge centrality for the entire sample
6. **Supplementary Table S4.** Centrality and expected influence for the network of high- and low-levels of childhood trauma.
7. **Supplementary Table S5.** Group comparison between male and female participants.
8. **Supplementary Table S6.** Centrality and expected influence for the network of male and female participants.
9. **The figure legend for Supplementary Figure S1.**

**Supplementary methods**

We conducted Pearson correlation test and partial correlation test (including age, education duration, and gender as covariates) between childhood trauma and other variates. Additionally, we conducted ANCOVA analysis for the group of high- and low-levels of childhood trauma, taking age, education duration, and gender into consideration. All above analysis were conducted in SPSS 21.0. Finally, as the independent sample t-test for gender group found significant differences between male and female participants in questionnaire scores, we conducted network comparison test for the gender group.

**Supplementary results**

Table S1 presented the Pearson’s correlation test between childhood trauma and other variates. In the partial correlation analysis, results were similar except for the correlation between emotional neglect in childhood trauma and fear of the pandemic became significant after controlling for education duration and/or gender. As for ANCOVA analysis, results were presented in Table S2. Group effects remain significant after controlling for age, education duration, and gender. The network comparison test made between the network of male and female participants showed no significant difference in global invariance (*p* = 0.726), network invariance (*p* = 0.206), and strength invariance test (*p* = 0.080).

**Supplementary Table S1.** Pearson’s correlations between childhood trauma and other variates for the entire sample.

|  | **CTQ-SF** | | | | | |
| --- | --- | --- | --- | --- | --- | --- |
|  | Total of CTQ-SF | CTQ1 | CTQ2 | CTQ3 | CTQ4 | CTQ5 |
| **FCV** | **.12^***^** | **.14^***^** | **.07^*^** | **.10^***^** | .05 | **.10^**^** |
| **PHQ** | **.31^***^** | **.37^***^** | **.19^***^** | **.16^***^** | **.27^***^** | **.25^***^** |
| **GAD** | **.23^***^** | **.32^***^** | **.12^***^** | **.08^**^** | **.21^***^** | **.19^***^** |
| **PCL** |  |  |  |  |  |  |
| Total of PCL | **.34^***^** | **.33^***^** | **.23^***^** | **.20^***^** | **.24^***^** | **.30^***^** |
| PCL1 | **.26^***^** | **.25^***^** | **.19^***^** | **.16^***^** | **.17^***^** | **.24^***^** |
| PCL2 | **.31^***^** | **.29^***^** | **.21^***^** | **.19^***^** | **.23^***^** | **.28^***^** |
| PCL3 | **.31^***^** | **.33^***^** | **.21^***^** | **.17^***^** | **.23^***^** | **.27^***^** |

*Note.* CTQ-SF = Childhood Trauma Questionnaire; CTQ1 = Emotional abuse; CTQ2 = Physical abuse; CTQ3 = Sexual abuse; CTQ4 = Emotional neglect; CTQ5 = Physical neglect; FCV = Fear of Coronavirus Disease 2019 (COVID-19) Scale; PHQ = Patient Health Questionnaire; GAD = Generalized Anxiety Disorder Questionnaire; PCL = Posttraumatic Stress Disorder Checklist-Civilian; PCL1 = Re-experiencing; PCL2 = Avoidance; PCL3 = Hyperarousal.

**Supplementary Table S2.** ANCOVA analysis between high- and low-levels of childhood trauma after taking age, education duration, and gender into consideration.

|  | High CTQ (*n* =125)  Mean (SE) | Low CTQ (*n* = 1122)  Mean (SE) | *F* | *p* | Effect size |
| --- | --- | --- | --- | --- | --- |
| **FCV** | 19.49 (.53) | 17.25 (.18) | 15.79 | **.000^***^** | 0.02 |
| **PHQ** | 18.47 (.41) | 15.39 (.14) | 50.71 | **.000^***^** | 0.05 |
| **GAD** | 14.27 (.38) | 11.78 (.13) | 38.20 | **.000^***^** | 0.04 |
| Total of PCL | 38.11 (.95) | 29.52 (.33) | 71.95 | **.000^***^** | 0.07 |
| PCL1: Re-experiencing | 11.13 (.34) | 8.71 (.12) | 45.63 | **.000^***^** | 0.04 |
| PCL2: Avoidance | 15.41 (.41) | 11.99 (.14) | 61.14 | **.000^***^** | 0.06 |
| PCL3: Hyperarousal | 11.56 (.34) | 8.83 (.12) | 57.99 | **.000^***^** | 0.06 |
| Total of CTQ-SF | 65.56 (.54) | 43.27 (.19) | 1505.32 | **.000^***^** | 0.61 |
| CTQ1: Emotional abuse | 12.08 (.20) | 6.44 (.07) | 728.52 | **.000^***^** | 0.43 |
| CTQ2: Physical abuse | 8.99 (.15) | 5.19 (.05) | 553.79 | **.000^***^** | 0.36 |
| CTQ3: Sexual abuse | 8.67 (.17) | 5.18 (.06) | 394.36 | **.000^***^** | 0.29 |
| CTQ4: Emotional neglect | 15.06 (.30) | 8.81 (.10) | 383.50 | **.000^***^** | 0.28 |
| CTQ5: Physical neglect | 12.15 (.22) | 7.02 (.08) | 483.63 | **.000^***^** | 0.33 |

*Note.* High CTQ = high-levels of childhood trauma; Low CTQ = low-levels of childhood trauma; SE = standard error; FCV = Fear of Coronavirus Disease 2019 (COVID-19) Scale; PHQ = Patient Health Questionnaire; GAD = Generalized Anxiety Disorder Questionnaire; PCL = Posttraumatic Stress Disorder Checklist-Civilian; CTQ-SF = Childhood Trauma Questionnaire.

**Supplementary Table S3.** Bridge centrality for the entire sample

|  | Bridge centrality | |
| --- | --- | --- |
|  | Strength | EI |
| **FCV** | 0.49 | 0.37 |
| **PHQ** | 0.41 | 0.41 |
| **GAD** | 0.44 | 0.26 |
| **PCL** |  |  |
| PCL1: Re-experiencing | 0.34 | 0.34 |
| PCL2: Avoidance | 0.22 | 0.22 |
| PCL3: Hyperarousal | 0.49 | 0.49 |
| **CTQ-SF** |  |  |
| CTQ1: Emotional abuse | 0.22 | 0.22 |
| CTQ2: Physical abuse | 0.10 | -0.04 |
| CTQ3: Sexual abuse | 0.09 | 0.01 |
| CTQ4: Emotional neglect | 0.11 | 0.03 |
| CTQ5: Physical neglect | 0.12 | 0.12 |

*Note.* EI = Expected Influence; FCV = Fear of Coronavirus Disease 2019 (COVID-19) Scale; PHQ = Patient Health Questionnaire; GAD = Generalized Anxiety Disorder Questionnaire; PCL = Posttraumatic Stress Disorder Checklist-Civilian; PCL1 = Re-experiencing; PCL2 = Avoidance; PCL3 = Hyperarousal; CTQ-SF = Childhood Trauma Questionnaire; CTQ1 = Emotional abuse; CTQ2 = Physical abuse; CTQ3 = Sexual abuse; CTQ4 = Emotional neglect; CTQ5 = Physical neglect.

**Supplementary Table S4.** Centrality and expected influence for the network of high- and low-levels of childhood trauma.

|  | High CTQ (*n* = 125) | | | |  | Low CTQ (*n* = 1122) | | | |
| --- | --- | --- | --- | --- | --- | --- | --- | --- | --- |
|  | Strength | Betweenness | Closeness | EI |  | Strength | Betweenness | Closeness | EI |
| **FCV** | -0.95 | -0.36 | 0.05 | -0.58 |  | -0.80 | -1.10 | -0.61 | -0.87 |
| **PHQ** | 0.67 | 0.74 | 1.03 | 0.71 |  | 0.97 | -0.94 | 0.96 | 0.98 |
| **GAD** | 0.36 | -0.91 | 0.65 | 0.46 |  | 0.81 | 0.47 | 0.89 | 0.82 |
| **PCL** |  |  |  |  |  |  |  |  |  |
| PCL1 | 1.26 | -0.47 | -0.12 | 1.18 |  | 1.00 | 0.31 | 0.39 | 1.01 |
| PCL2 | 0.60 | -0.91 | -0.31 | 0.65 |  | 1.07 | 1.57 | 0.98 | 1.08 |
| PCL3 | 1.03 | 0.30 | 0.46 | 0.99 |  | 0.97 | 1.57 | 1.18 | 0.97 |
| **CTQ-SF** |  |  |  |  |  |  |  |  |  |
| CTQ1 | 0.09 | 1.74 | 1.11 | 0.24 |  | -0.25 | 0.78 | 0.12 | -0.23 |
| CTQ2 | 0.38 | 1.63 | 0.73 | 0.38 |  | -1.15 | -0.63 | -1.14 | -1.12 |
| CTQ3 | -0.21 | 0.08 | -0.10 | -0.84 |  | -1.58 | -1.10 | -1.94 | -1.55 |
| CTQ4 | -1.44 | -0.91 | -1.35 | -1.92 |  | -0.17 | -0.31 | -0.35 | -0.24 |
| CTQ5 | -1.79 | -0.91 | -2.14 | -1.25 |  | -0.88 | -0.63 | -0.48 | -0.85 |

*Note.* High CTQ = high-levels of childhood trauma; Low CTQ = low-levels of childhood trauma; EI = Expected Influence; FCV = Fear of Coronavirus Disease 2019 (COVID-19) Scale; PHQ = Patient Health Questionnaire; GAD = Generalized Anxiety Disorder Questionnaire; PCL = Posttraumatic Stress Disorder Checklist-Civilian; PCL1 = Re-experiencing; PCL2 = Avoidance; PCL3 = Hyperarousal; CTQ-SF = Childhood Trauma Questionnaire; CTQ1 = Emotional abuse; CTQ2 = Physical abuse; CTQ3 = Sexual abuse; CTQ4 = Emotional neglect; CTQ5 = Physical neglect.

**Supplementary Table S5.** Group comparison between male and female participants.

|  | Male (*n* = 361) | Female (*n* = 886) | *t* | *p* | Cohen’s *d* |
| --- | --- | --- | --- | --- | --- |
| **FCV** | 16.64 | 17.66 | -2.76 | **.006^**^** | 0.19 |
| **PHQ** | 15.32 | 15.80 | -1.71 | .088 | 0.11 |
| **GAD** | 11.48 | 12.24 | -3.14 | **.002^**^** | 0.20 |
| **PCL** |  |  |  |  |  |
| Total of PCL | 31.81 | 29.87 | 2.83 | **.005^**^** | 0.19 |
| PCL1: Re-experiencing | 9.17 | 8.80 | 1.56 | .120 | 0.10 |
| PCL2: Avoidance | 13.07 | 12.08 | 3.33 | **.001^**^** | 0.23 |
| PCL3: Hyperarousal | 9.56 | 8.98 | 2.44 | **.015^*^** | 0.16 |
| **CTQ-SF** |  |  |  |  |  |
| Total of CTQ-SF | 46.91 | 44.77 | 3.47 | **.001^**^** | 0.25 |
| CTQ1: Emotional abuse | 6.98 | 6.99 | -.09 | .932 | 0.01 |
| CTQ2: Physical abuse | 5.97 | 5.38 | 3.95 | **.000^***^** | 0.33 |
| CTQ3: Sexual abuse | 5.88 | 5.35 | 3.39 | **.001^**^** | 0.29 |
| CTQ4: Emotional neglect | 9.78 | 9.32 | 2.02 | **.049^*^** | 0.13 |
| CTQ5: Physical neglect | 7.96 | 7.25 | 3.92 | **.000^***^** | 0.27 |

*Note.* FCV = Fear of Coronavirus Disease 2019 (COVID-19) Scale; PHQ = Patient Health Questionnaire; GAD = Generalized Anxiety Disorder Questionnaire; PCL = Posttraumatic Stress Disorder Checklist-Civilian; CTQ-SF = Childhood Trauma Questionnaire.

**Supplementary Table S6.** Centrality and expected influence for the network of male and female participants.

|  | Males (*n* = 361) | | | |  | Females (*n* = 886) | | | |
| --- | --- | --- | --- | --- | --- | --- | --- | --- | --- |
|  | Strength | Betweenness | Closeness | EI |  | Strength | Betweenness | Closeness | EI |
| **FCV** | -2.07 | -1.37 | -1.73 | -2.01 |  | -2.04 | -1.27 | -1.91 | -2.04 |
| **PHQ** | 0.24 | 1.00 | 1.29 | 0.30 |  | 0.76 | -0.41 | 0.84 | 0.76 |
| **GAD** | 0.37 | 0.86 | 1.14 | 0.09 |  | 0.26 | -0.62 | 0.66 | 0.26 |
| **PCL** |  |  |  |  |  |  |  |  |  |
| PCL1 | 0.80 | -0.11 | -0.30 | 0.86 |  | 0.85 | 0.66 | -0.08 | 0.85 |
| PCL2 | 0.98 | 1.14 | 0.53 | 1.04 |  | 0.95 | 0.02 | 0.82 | 0.95 |
| PCL3 | 0.38 | 0.72 | 0.78 | 0.44 |  | 0.54 | 1.09 | 1.27 | 0.54 |
| **CTQ-SF** |  |  |  |  |  |  |  |  |  |
| CTQ1 | 0.92 | 0.86 | 0.63 | 0.98 |  | 1.01 | 2.17 | 0.97 | 1.01 |
| CTQ2 | 0.61 | -0.25 | 0.03 | 0.59 |  | 0.13 | 0.02 | -0.47 | 0.13 |
| CTQ3 | -0.49 | -1.37 | -0.82 | -0.69 |  | -1.12 | -1.05 | -0.94 | -1.12 |
| CTQ4 | -1.51 | -1.37 | -1.40 | -1.44 |  | -0.46 | 0.02 | -0.30 | -0.46 |
| CTQ5 | -0.22 | -0.11 | -0.15 | -0.16 |  | -0.88 | -0.62 | -0.87 | -0.88 |

*Note.* EI = Expected Influence; FCV = Fear of Coronavirus Disease 2019 (COVID-19) Scale; PHQ = Patient Health Questionnaire; GAD = Generalized Anxiety Disorder Questionnaire; PCL = Posttraumatic Stress Disorder Checklist-Civilian; PCL1 = Re-experiencing; PCL2 = Avoidance; PCL3 = Hyperarousal; CTQ-SF = Childhood Trauma Questionnaire; CTQ1 = Emotional abuse; CTQ2 = Physical abuse; CTQ3 = Sexual abuse; CTQ4 = Emotional neglect; CTQ5 = Physical neglect.

**Fig. S1. A** Network visualization of male participants (*n* = 361). **B** Network visualization of female participants (*n* = 886). The gray node represented the fear of COVID-19. The light blue nodes represented depression and anxiety. The green nodes were a combination of traumatic symptoms post COVID-19. Symptoms of childhood trauma were represented by the yellow nodes. Blue lines were positive correlations between nodes and red lines were negative correlations. The thicker and shorter the lines, the stronger and closer connections. FCV = Fear of Coronavirus Disease 2019 (COVID-19) Scale; PHQ = Patient Health Questionnaire; GAD = Generalized Anxiety Disorder Questionnaire; PCL = Posttraumatic Stress Disorder Checklist-Civilian; CTQ = Childhood Trauma Questionnaire. **C** Standardized centrality indices plot, including strength, betweenness, closeness, and expected influence, of the two networks. The red line represented male participants and the blue line represented female participants.
