# Supplementary figures and images for: Relationships between childhood trauma and mental health during the COVID-19 pandemic: a network analysis
Source: Front Psychiatry. 2023 Sep 8;14:1251473. doi: 10.3389/fpsyt.2023.1251473 (PMC10515217; doi:10.3389/fpsyt.2023.1251473)

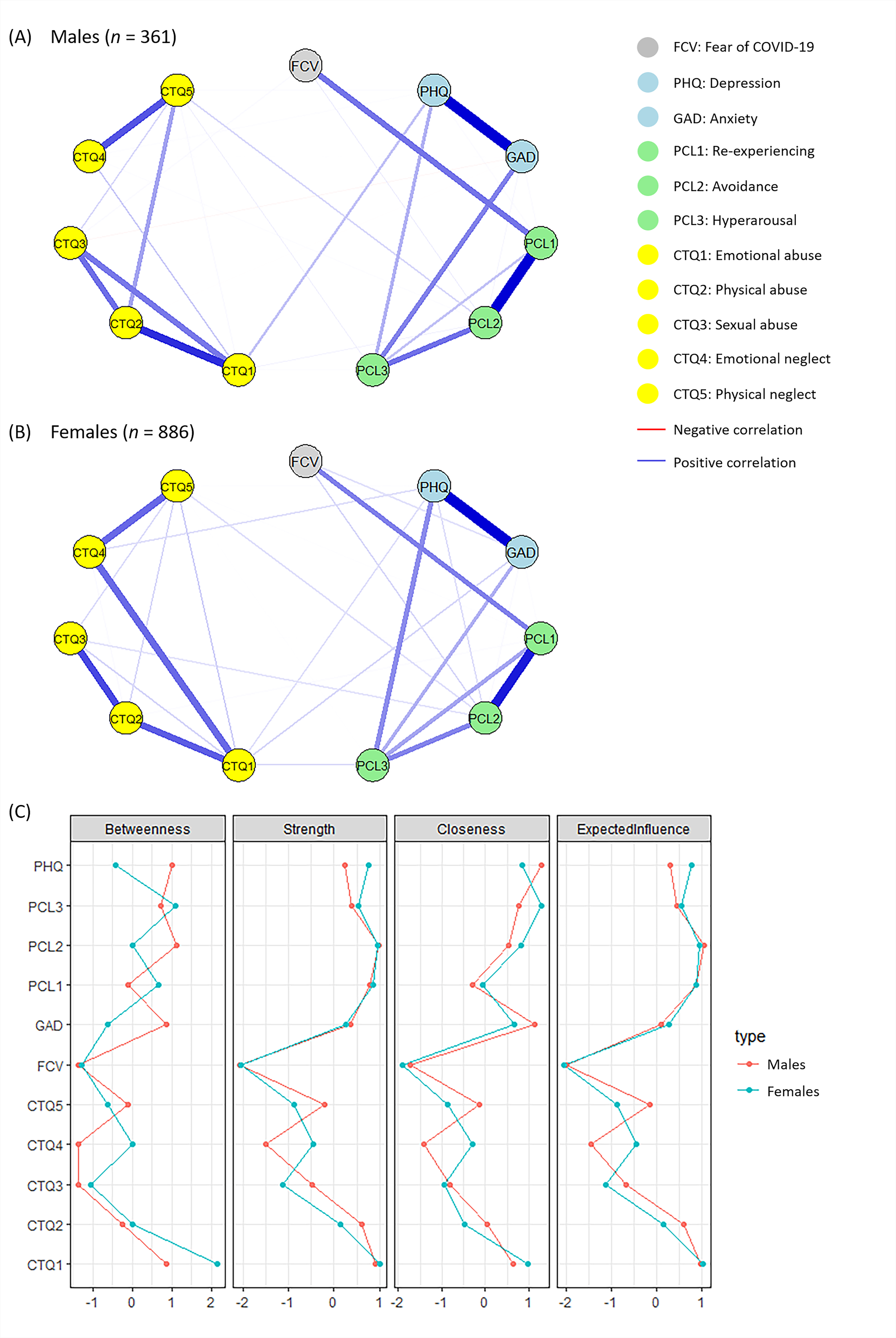

Supplement: Supplementary file 2 [file Image_1.TIF]
